# Is the 10‐Year Trajectory of Physical Activity Associated With the Incidence of Mild Cognitive Impairment in Older Adults?

**DOI:** 10.1111/psyg.70141

**Published:** 2026-01-29

**Authors:** Joel de Almeida Siqueira Junior, Francisco Timbó de Paiva Neto, Carla Elane Silva Santos, Lucas Gomes Alves, Eleonora d'Orsi, Cassiano Ricardo Rech

**Affiliations:** ^1^ Graduate Program in Physical Education Federal University of Santa Catarina Florianópolis Brazil; ^2^ Hospital Israelita Albert Einstein São Paulo Brazil; ^3^ Graduate Program in Collective Health Federal University of Santa Catarina Florianópolis Brazil

**Keywords:** aging, cognition, epidemiology, physical activity, trajectory

## Abstract

**Objective:**

To identify leisure‐time physical activity trajectories in older adults and examine their association with the incidence of mild cognitive impairment (MCI) over a decade.

**Methods:**

This longitudinal study used data from the EpiFloripa Aging Cohort Study (2009–2019). The sample included older adults of both sexes, aged 60 years or older, residing in Florianópolis, Santa Catarina, Brazil. MCI was assessed using the Mini‐Mental State Examination, and incidence was defined as new cases identified after baseline. Leisure‐time physical activity was measured using the International Physical Activity Questionnaire. Older adults reporting no participation (≤ 10 min/week) in moderate‐to‐vigorous physical activity (MVPA) or walking were classified as following an ‘inactive trajectory’, while those maintaining ≥ 10 min/week of MVPA or walking were considered to follow an ‘active trajectory’. Incidence rate ratios (IRR) and their 95% confidence intervals (95% CI) were calculated to test the associations.

**Results:**

A total of 731 older adults were followed (66.6% women). The incidence of MCI was 104 new cases (18.5%; 95% CI: 14.8–20.9). Older adults who maintained an active trajectory of MVPA and walking in leisure time had a 40% lower risk (IRR: 0.60; 95% CI: 0.38–0.92) and a 34% lower risk (IRR: 0.76; 95% CI: 0.47–0.96), respectively, of developing MCI during the follow‐up period.

**Conclusion:**

Maintaining an active trajectory of physical activity was associated with a reduced risk of developing MCI after 10 years of follow‐up. Leisure‐time MVPA and walking programs may represent promising strategies to promote cognitive function in older adults.

## 1 Introduction

The rapid growth of the aging population has emerged as one of the most significant demographic transformations, with a substantial increase in the proportion of older adults [1]. Projections indicate that by 2050, Brazil may reach 47% of individuals aged 60 years or older [2]. This shift poses increasing challenges to public health systems, requiring the implementation of strategies that not only extend life expectancy but also promote healthy aging [3]. Within this context, preserving cognitive health is a key component for ensuring quality of life and independence in advanced age [4].

Mild cognitive impairment (MCI) is a clinical condition that lies between expected cognitive aging and dementia, such as Alzheimer's disease, and affects approximately 19.7% of people over 50 years old [5, 6]. MCI is characterised by impairments in memory, learning, language, orientation and executive functions that become more evident during aging, and it is associated with increased risk of reduced autonomy and independence [7]. Identifying modifiable factors that may prevent or delay the onset of MCI is therefore a promising strategy for the development of public health policies, programs and interventions [8].

Among the potentially protective factors against the incidence of MCI, regular physical activity has consistently stood out in the scientific evidence [9]. Multiple systematic reviews have demonstrated the beneficial effects of physical activity, including walking, on MCI in older adults [10, 11], suggesting mechanisms ranging from increased cerebral blood flow to the release of neurotrophins, such as brain‐derived neurotrophic factor, which enhance synaptic plasticity and neurogenesis [12]. Other findings highlight significant improvements in memory, learning, language, orientation, executive functions, processing speed, autonomy and functional independence [13, 14].

The use of trajectory analysis to quantify brief periods of physical activity, such as 10 min, is grounded in robust evidence demonstrating clinically meaningful benefits of minimal exercise doses in geriatric populations. Recent studies indicate that any amount of moderate‐to‐vigorous physical activity (MVPA) is associated with a reduced risk of all‐cause dementia, regardless of frailty status [15], while minimal exercise amounts, defined as walking for approximately 15–30 min performed daily or weekly, have shown significant preventive effects on dementia incidence among older adults with osteoarthritis [16]. Additionally, walking interventions have produced significant improvements in aerobic capacity and endurance in individuals with MCI [17]. Thus, monitoring physical activity patterns within short temporal windows using trajectory analysis allows the capture of behaviour changes that are relevant to cognitive and functional outcomes, constituting a methodologically appropriate approach for evaluating low‐intensity interventions in older populations.

Despite the robustness of these findings, most available investigations examine physical activity and walking as static variables, often assessed at a single point in time. This approach overlooks physical activity trajectories throughout the aging process, which may exert different influences on health outcomes, including cognitive health [18]. In this sense, longitudinal studies tend to assess physical activity only at baseline and at the end of follow‐up; therefore, analysing trajectories may provide a clearer understanding of the association with the risk of developing MCI [19, 20].

Therefore, the aim of this study was to identify leisure‐time physical activity and walking trajectories in a representative sample of Brazilian older adults followed for a decade and to examine the association between these trajectories and the incidence of MCI.

## 2 Methods

### 2.1 Study Design and Ethical Aspects

This population‐based longitudinal analysis was conducted using 10‐year follow‐up data (2009–2019) from the EpiFloripa Aging Cohort Study (https://epifloripaidoso.paginas.ufsc.br), carried out in Florianópolis, a city located in southern Brazil. The city is predominantly situated on an island (97.2%), has a higher Human Development Index compared to the national average (0.847 vs. 0.759) [21] and a moderate social inequality index (0.54) [22]. Florianópolis also offers well‐maintained public open spaces with diverse infrastructure for physical activity [23].

Details of the study design and sampling procedures have been previously published [24, 25]. Briefly, the sample consisted of older adults (≥ 60 years) of both sexes, residing in the urban area of the city. Institutionalised older adults were excluded from the study. Face‐to‐face interviews were conducted at participants' homes by trained interviewers using a structured computer‐assisted questionnaire. Interview quality control was performed through the administration of a reduced questionnaire to 15% of the participants, and Kappa values were considered satisfactory for ensuring adequate data quality.

**FIGURE 1:**
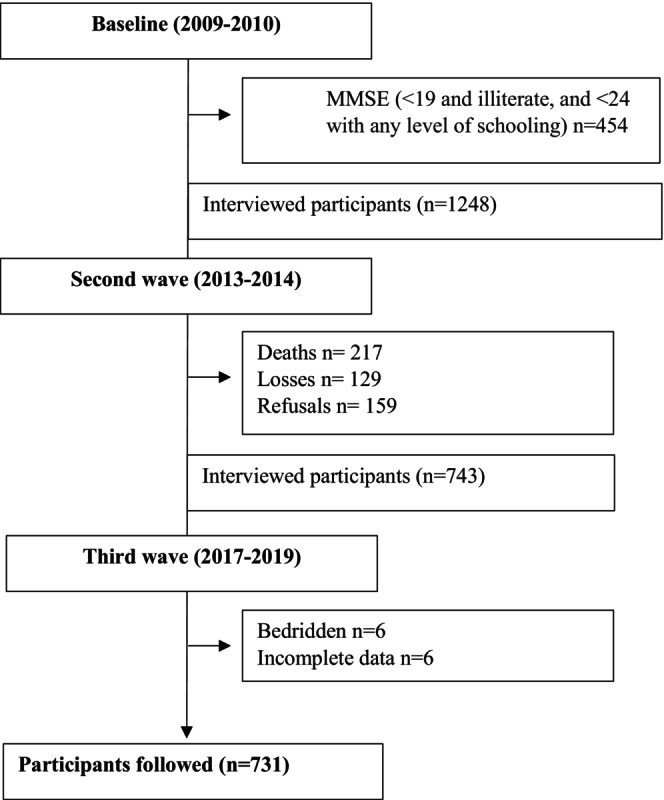
Flowchart describing the sampling plan. EpiFloripa Aging Cohort Study. Florianópolis, Santa Catarina, Brazil, 2009/2019 (*n* = 731).

The incidence analysis of MCI was conducted only among older adults without this condition at baseline. Specifically, 454 participants were excluded because they scored < 19 on the Mini‐Mental State Examination (MMSE) if illiterate, or < 24 if they had any level of schooling. All older adults who participated at baseline (2009–2010, *n* = 1702) and in the second wave (2013–2014, *n* = 1248) were invited to take part in the third wave (2017–2019, *n* = 743). Repeated measures from all three waves were included in the analysis (Figure 1).

The study was submitted to and approved by the Research Ethics Committee of the Federal University of Santa Catarina. Ethical approval was granted for the baseline (No. 352/2008), the second wave (No. 16731313.0.0000.0121) and the third wave (No. 1.957.977). All participants voluntarily consented to take part in the study and signed an informed consent form.

### 2.2 Outcome

The MMSE, consisting of 30 items, was used to screen for the probable presence of MCI. The MMSE assesses different cognitive domains, such as temporal and spatial orientation, short‐term memory, calculation, comprehension and writing [26]. We adopted the cut‐off points suggested by Almeida [27], which indicate probable MCI for individuals with scores < 19 points and no formal education, and < 24 points for those with some level of education. In the end, older adults were classified dichotomously according to the ‘presence’ or ‘absence’ of MCI. The instrument gained wide popularity in epidemiological studies and was validated in Brazil, showing a sensitivity of 80.0% and specificity of 70.9% for detecting MCI among illiterate older adults, and 77.8% sensitivity and 75.4% specificity, respectively, for those with some level of education [27].

### 2.3 Exposure

Leisure‐time MVPA and walking during the previous 7 days of a typical week were assessed using the extended version of the International Physical Activity Questionnaire (IPAQ), validated for Brazilian older adults [28, 29]. In this study, information on moderate‐ and vigorous‐intensity activities was combined into a single variable, calculated according to the World Health Organization physical activity recommendations, in which vigorous‐intensity activity was weighted as half of moderate‐intensity activity (moderate activity + vigorous activity × 2) [30].

MVPA and walking trajectories were defined as descriptive patterns of activity status across repeated assessments over a 10‐year follow‐up, based on time spent in these activities at each observation period. Older adults who did not engage in physical activity (≤ 10 min/week) during the period were classified as having an inactive trajectory, while those who engaged in ≥ 10 min/week in at least two observation periods were classified as having an active trajectory. This minimum threshold corresponds to the IPAQ criterion for considering valid activity episodes and was applied to both walking and MVPA solely for exposure classification purposes, without implying equivalence in intensity or physiological effects [28, 29, 31].

### 2.4 Covariates

Sociodemographic factors included sex (female and male), age group (60–69, 70–79 and 80 years or older) and skin colour (White and non‐White). Depressive symptoms were assessed using the Geriatric Depression Scale (GDS), with scores ≥ 6 points indicating depressive symptomatology [32]. Depressive symptoms were treated as a baseline confounder and included as an adjustment variable to control for initial differences between participants, as they may temporally precede both longitudinal physical activity patterns and the incidence of MCI.

### 2.5 Data Analysis

Descriptive analyses were expressed as absolute and relative frequencies. The distributions of covariates according to MCI incidence were determined using Pearson's chi‐square test.

To estimate the incidence of MCI, person‐years were calculated for the period in which older adults were at risk of developing MCI within the cohort. Cumulative incidence was then obtained by dividing the number of new cases identified during the follow‐up by the number of participants without the condition at baseline, multiplying this value by 1000, and dividing by the average person‐years [33].

Crude and adjusted incidence rate ratios (IRR), adjusted for age group, education, skin colour and depressive symptoms, along with their 95% confidence intervals (95% CI), were used to test the association between MVPA and walking trajectories and the incidence of MCI. Statistical significance was set at *p* < 0.05. Analyses were conducted using Stata/SE 14.0 (Stata Corp., College Station, United States).

## 3 Results

Table 1 presents the characteristics of participants across the three waves and the incidence of MCI. Over the 10‐year period, 731 older adults were followed (66.6% women), aged between 67 and 108 years. The incidence of MCI from baseline to the end of follow‐up was 104 new cases (18.5%; 95% CI: 14.8–20.9), with 55 new cases (9.3%; 95% CI: 7.2–11.9) identified in Wave 2 and 49 new cases (9.2%; 95% CI: 7.0–11.9) identified in Wave 3. The mean follow‐up time in the cohort was 8.1 years (SD = 7.0), and the cumulative incidence of MCI was 21 cases per 1000 person‐years.

**TABLE 1:** Characteristics of the participants of the EpiFloripa Aging Cohort Study. Florianópolis, Santa Catarina, Brazil, 2009/2019 (*n* = 731).

Variables	Category	MCI incidence^a^		*p*
		*n* (%)	*χ* ^2,^ ^b^	
Sex	Female	77 (20.1)	4.61	0.032
	Male	27 (13.0)		
Age group	60–69 years	47 (12.4)	31.9	< 0.001
	70–79 years	44 (23.5)		
	≥ 80 years	13 (52.0)		
Skin colour^c^	White	81 (15.8)	10.4	0.001
	Non‐White	23 (31.1)		
Education level	0–4 years	88 (29.7)	62.0	< 0.001
	5–8 years	7 (6.4)		
	≥ 9 years	9 (4.4)		
Depressive symptoms^d^	Absence	78 (15.9)	8.33	0.004
	Presence	26 (28.7)		

MCI incidence	Wave 2		Wave 3	
	*n* (%)	95% CI	*n* (%)	95% CI
Presence	55 (9.3)	7.2–11.9	49 (9.2)	7.0–11.9

^a^ Incidence of mild cognitive impairment assessed by the Mini‐Mental State Examination (MMSE).

^b^ Pearson *χ* ^2^.

^c^ Non‐White skin color (self‐reported as Black, Brown, Yellow, or Indigenous).

^d^ Depressive symptoms assessed by the Geriatric Depression Scale (GDS).

The incidence of MCI was significantly higher among women (20.1%; *p* = 0.032), participants aged > 80 years (52.0%; *p* < 0.001), those reporting non‐White skin colour (31.1%; *p* = 0.001), individuals with 0–4 years of schooling (29.7%; *p* < 0.001) and those presenting depressive symptoms (28.7%; *p* = 0.004). Baseline sociodemographic characteristics and depressive symptoms stratified by MVPA and walking trajectories are presented in Table S1.

**FIGURE 2:**
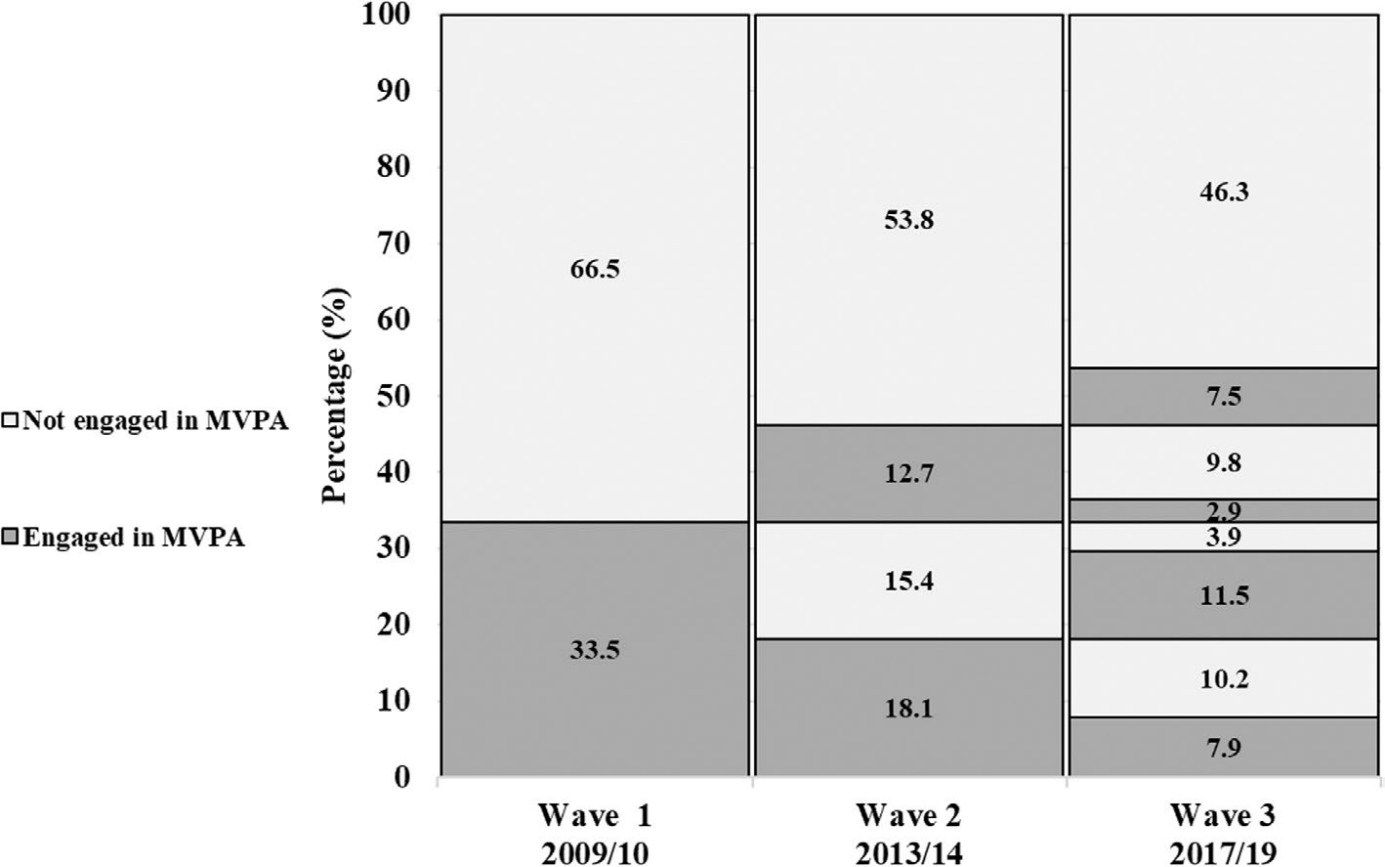
Trajectory of moderate‐to‐vigorous physical activity in the leisure domain among participants of the EpiFloripa Aging Cohort Study. Florianópolis, Santa Catarina, Brazil, 2009–2019 (*n* = 731).

**FIGURE 3:**
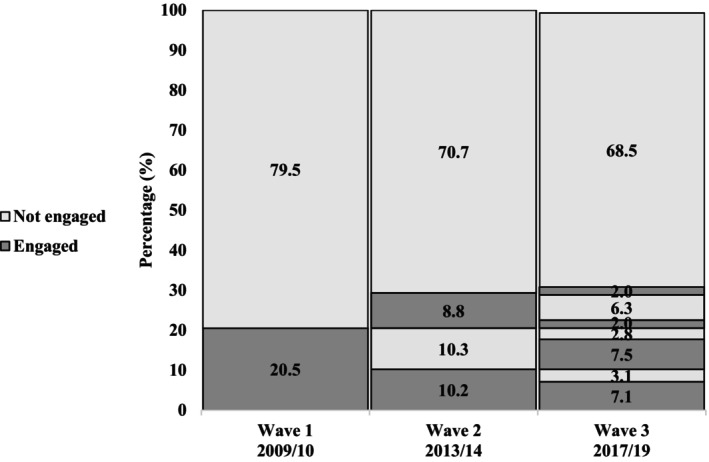
Trajectory of walking in the leisure domain among participants of the EpiFloripa Aging Cohort Study. Florianópolis, Santa Catarina, Brazil, 2009–2019 (*n* = 731).

Figures 2 and 3 show the proportions of leisure‐time walking and MVPA (≥ 10 min). About 33.5% of older adults engaged in MVPA at baseline. This proportion decreased by 2.7% between 2009 and 2013 and reached 29.8% in 2019. Regarding walking, only 20.5% reported participation at baseline, with a 1.5% reduction during follow‐up and prevalence remained stable in the third wave at 18.6%.

Analysis of the relationship between MCI incidence and MVPA and walking trajectories (engagement in at least two follow‐up periods) showed that older adults who maintained an active trajectory of MVPA and walking in leisure time had a 40% lower risk (IRR: 0.60; 95% CI: 0.38–0.92) and a 34% lower risk (IRR: 0.76; 95% CI: 0.47–0.96), respectively, of developing MCI during the follow‐up period compared with those with inactive trajectories (Table 2). Sensitivity analyses using the WHO‐recommended cutoff of ≥ 150 min/week for MVPA and walking are presented in Table S2.

**TABLE 2:** Crude and adjusted analysis of the trajectories of moderate‐to‐vigorous physical activity and walking in the leisure domain with the incidence of Mild Cognitive Impairment among participants of the EpiFloripa Aging Cohort Study. Florianópolis, Santa Catarina, Brazil, 2009–2019 (*n* = 731).

Variables	Category	*n* (%)	MCI incidence	
			Crude analysis	Adjusted analysis^a^
			IRR (95% CI)	IRR (95% CI)
MVPA trajectory	Inactive	75 (21.1)	1	1
	Active	29 (12.1)	0.57 (0.37–0.88)*	0.60 (0.38–0.92)*
Walking trajectory	Inactive	81 (18.8)	1	1
	Active	23 (6.3)	0.68 (0.42–0.94)*	0.76 (0.47–0.96)*

Abbreviations: 95% CI, confidence interval; IRR, incidence rate ratio.

^a^ Adjusted for age group, education level, skin colour and depressive symptoms.

* *p* < 0.05.

## 4 Discussion

The aim of this study was to identify leisure‐time physical activity trajectories in older adults and examine their association with the incidence of MCI over a decade. Using data spanning 10years, we found that approximately 33.5% of older adults engaged in MVPA at baseline. This proportion decreased by 2.7% between 2009 and 2013, reaching 29.8% in 2019. Regarding walking, only 20.5% of participants reported time spent in this activity at baseline, which decreased by 1.5% during follow‐up and remained stable in the third wave at 18.6%. Furthermore, we observed that older adults who maintained an active trajectory of MVPA and walking had a lower risk of developing MCI during the follow‐up period compared with those with inactive trajectories.

Concerning sex, the findings suggest a greater impact of MCI among older women. The incidence of MCI was significantly higher among women (20.1%; *p* = 0.032), highlighting the need for gender‐specific approaches in screening and prevention strategies for MCI in the older population. The higher proportion of women among older adults has important public health implications, as women tend to have a higher prevalence of chronic conditions and greater demand for health services and long‐term care [34]. Therefore, it is essential that health services address the specific health needs of older women while also developing strategies to increase engagement and care for the male older population.

The findings indicate that the incidence of MCI was significantly higher among participants reporting non‐White skin colour, with an incidence rate of 31.1% (*p* = 0.001). This result aligns with existing literature indicating racial disparities in cognitive health, often attributed to differences in socioeconomic status, unequal access to healthcare, education levels and exposure to life‐course risk factors, such as less favourable living and working conditions [35, 36]. Individuals with non‐White skin colour may experience a greater cumulative burden of stress and social adversity, which has been associated with an increased risk of MCI [37]. Accordingly, it is essential for health managers and policymakers to develop strategies that train professionals to recognise and address these ethnic‐racial particularities.

Moreover, lower educational attainment, also observed as a significant risk factor for MCI (29.7%; *p* < 0.001), may interact with socioeconomic conditions to exacerbate cognitive health inequalities [38]. These results underscore the urgent need for public health strategies and interventions that address racial disparities and promote equity in cognitive health, ensuring that vulnerable populations have access to quality educational resources and healthcare throughout the life course.

Older adults with an active trajectory of leisure‐time MVPA exhibited a lower risk of developing MCI over a decade. Regular physical activity and cognitive performance are strongly associated, and maintaining an active lifestyle during aging results in reduced MCI incidence [39, 40]. Accordingly, longitudinal studies with follow‐up periods similar to the present study have investigated the association between MVPA trajectories and cognitive function in older adults, finding that greater declines in regular physical activity increase the risk of developing MCI [41, 42]. Thus, improvements in cognition through an active leisure‐time MVPA trajectory may be related to greater social interaction among older adults. Achieving recommended levels of physical activity is believed to enhance cognitive domains such as attention and functionality and may even reverse episodes of MCI [43]. In summary, the findings suggest that expanding access to public leisure spaces and providing facilities for group MVPA activities could support the maintenance of active behaviour and represent an important collective‐level health promotion strategy.

Regarding walking, older adults who maintained an active leisure‐time walking trajectory also exhibited a lower risk of developing MCI over a decade. Previous studies have shown that older adults engaged in moderate‐to‐vigorous leisure‐time walking experience cognitive benefits, including improvements in memory and spatial orientation [44, 45]. However, the benefits of an active walking trajectory in older adults may also be linked to reduced social isolation and enhanced social support [46]. Moreover, walking appears to function as an important mediator within the context of social and environmental determinants of health and serves as a preventive component that reduces the incidence of MCI in older populations [47]. These results highlight the need to promote public health policies that implement walking programs for older adults, particularly in well‐planned urban spaces, to facilitate socialisation and maximise positive impacts on cognitive health over time. This approach underscores the value of low‐cost interventions and the importance of fostering preventive strategies for healthy aging.

This study has several scientific strengths that contribute significantly to understanding the incidence of MCI in older adults. The population‐based longitudinal design allowed participants to be followed over a decade, capturing changes over time and enabling robust analyses of physical activity trajectories and their association with MCI incidence. Additionally, the use of validated instruments and standardised methods, such as the MMSE for cognitive assessment and the IPAQ for measuring physical activity, ensured the consistency and reliability of the collected data. The sample of older adults was representative of the city of Florianópolis, a region with a high quality of life and socioeconomic diversity, providing a solid basis for generalisations within the Brazilian context. Furthermore, rigorous control of confounding variables, including age, education, skin colour and depressive symptoms, strengthened the internal validity of the observed associations. These robust methodological aspects not only support the study's conclusions but also offer opportunities for improvement in future research and interventions aimed at promoting cognitive health in the older population.

However, the findings should be interpreted considering certain limitations. The MMSE is a screening instrument rather than a diagnostic tool; therefore, the results should not be interpreted as evidence of clinically defined MCI. Physical activity was assessed by self‐report, which is subject to recall and social desirability bias and may have resulted in exposure misclassification. Although the longitudinal design strengthens temporal inference, reverse causation cannot be fully excluded, as early cognitive decline may precede reductions in physical activity. Attrition during follow‐up may have introduced selection bias, potentially affecting incidence estimates if participants lost to follow‐up differed systematically in cognitive status or physical activity patterns. While key confounders, including depressive symptoms and age, were controlled at baseline, changes in these factors over time were not captured and may have influenced both physical activity and cognitive outcomes. These limitations should be considered when interpreting the observed associations and highlight the need for future studies using objective measures, repeated assessments and time‐dependent modelling to better clarify causal pathways.

## 5 Conclusion

The findings of this study indicate that older adults who maintained an active trajectory of leisure‐time physical activity (either MVPA or walking) had a lower risk of developing MCI over a decade. Each activity domain was evaluated independently, and the observed associations suggest that sustained engagement in different forms of physical activity may support cognitive health in later life. Importantly, these results provide practical insights for the design of future initiatives, interventions and public policies to promote physical activity among older adults in the Global South, where structural barriers, social inequalities and limited access to formal exercise settings remain significant challenges. Strategies that emphasize accessible, context‐sensitive and achievable forms of physical activity may be particularly relevant for supporting healthy aging and reducing the burden of cognitive impairment in these settings.

## Funding

The EpiFloripa Aging Cohort Study received support from different sources across its waves. Wave 1 was funded by the Brazilian National Council for Scientific and Technological Development (CNPq) (Grant 569834/2008‐2). Wave 2 had no specific funding and was conducted through partnerships with UFSC, other projects and the involvement of students and professors, with infrastructure provided by UFSC and netbooks supplied by the Oswaldo Cruz Foundation (FIOCRUZ). Wave 3 was funded by the Economic and Social Research Council (ESRC) of the United Kingdom through the Multicenter Promoting Independence in Dementia (PRIDE) project (contract ESRC‐UK/UFSC/FAPEU 75/2017). The funders had no role in study design, data collection, analysis, interpretation, manuscript preparation or publication decisions. In addition, J.A.S.J. received support from the Coordination for the Improvement of Higher Education Personnel (CAPES), Brazil—Financing Code 001, and L.G.A. received support from the Research and Innovation Support Foundation of the State of Santa Catarina (Fundação de Amparo à Pesquisa e Inovação do Estado de Santa Catarina—FAPESC), Edital 19/2024.

## Conflicts of Interest

The authors declare no conflicts of interest.

## Supporting information


**Table S1:** Association between MVPA and walking trajectory in the participants of the EpiFloripa Aging Cohort Study. Florianópolis, Santa Catarina, Brazil, 2009/2019 (*n* = 731).

**Table S2:** Characteristics of the participants of the EpiFloripa Aging Cohort Study. Florianópolis, Santa Catarina, Brazil, 2009/2019 (*n* = 731).

## Data Availability

The data that support the findings of this study are available on request from the corresponding author. The data are not publicly available due to privacy or ethical restrictions.
