# Supplementary material for: Is the 10‐Year Trajectory of Physical Activity Associated With the Incidence of Mild Cognitive Impairment in Older Adults?
Source: Psychogeriatrics. 2026 Jan 29;26(2):e70141. doi: 10.1111/psyg.70141 (PMC12854854; doi:10.1111/psyg.70141)
Supplement: Supplementary file 1 — Table S1: Association between MVPA and walking trajectory in the participants of the EpiFloripa Aging Cohort Study. Florianópolis, Santa Catarina, Brazil, 2009/2019 (n = 731). [file PSYG-26-0-s002.docx]

| **Table S1.** Association between MVPA and walking trajectory in the participants of the EpiFloripa Aging Cohort Study. Florianópolis, Santa Catarina, Brazil, 2009/2019 (n = 731) | | | | |
| --- | --- | --- | --- | --- |
| **Variables** | **Category** | **MCI Incidence**^†^ | | **p-valor** |
|  |  | **n (%)** | **χ^2^** |  |
| **MVPA** **Trajectory*** | Inactive (n=393) | 75 (19.1) | 1.79 | 0.181 |
|  | Active (n=198) | 29 (14.6) |  |  |
|  |  |  |  |  |
| **Walking Trajectory*** | Inactive (n=527) | 95 (18.0) | 2.30 | 0.130 |
|  | Active (n=59) | 6 (10.2) |  |  |
| ^†^ Incidence of mild cognitive impairment assessed by the Mini-Mental State Examination (MMSE).  *Engage in physical activity and walking ≤150 min/week.  MVPA = moderate-to-vigorous physical activity  χ² = Pearson Chi-square. | | | | |
