# Supplementary material for: Is the 10‐Year Trajectory of Physical Activity Associated With the Incidence of Mild Cognitive Impairment in Older Adults?
Source: Psychogeriatrics. 2026 Jan 29;26(2):e70141. doi: 10.1111/psyg.70141 (PMC12854854; doi:10.1111/psyg.70141)
Supplement: Supplementary file 2 — Table S2: Characteristics of the participants of the EpiFloripa Aging Cohort Study. Florianópolis, Santa Catarina, Brazil, 2009/2019 (n = 731). [file PSYG-26-0-s001.docx]

| **Table S2.** Characteristics of the participants of the EpiFloripa Aging Cohort Study. Florianópolis, Santa Catarina, Brazil, 2009/2019 (n = 731) | | | | | | | |
| --- | --- | --- | --- | --- | --- | --- | --- |
| **Variables** | **Category** | **MVPA**  **Trajectory**^†^ | | **p-value** | **Walking**  **Trajectory**^†^ | | **p-value** |
|  |  | **n (%)** | **x²*** |  | **n (%)** | **x²*** |  |
| **Sex** | Female | 114 (29.7) | 7.16 | **0,007** | 26 (6.9) | 12.2 | **<0.001** |
|  | Male | 84 (40.6) |  |  | 33 (15.9) |  |  |
|  |  |  |  |  |  |  |  |
| **Age group** | 60 to 69 years | 128 (33.9) | 1.05 | 0.589 | 39 (10.4) | 0.19 | 0.907 |
|  | 70 to 79 years | 64 (34.0) |  |  | 18 (9.6) |  |  |
|  | > 80 years | 6 (24.0) |  |  | 2 (8.0) |  |  |
|  |  |  |  |  |  |  |  |
| **Skin color^a^** | White | 174 (33.8) | 0.05 | 0.818 | 52 (10.2) | 0.02 | 0.876 |
|  | Non-white | 24 (32.4) |  |  | 7 (9.6) |  |  |
|  |  |  |  |  |  |  |  |
| **Education level** | 0 a 4 years | 79 (26.6) | 16.5 | **<0.001** | 19 (6.5) | 8.55 | **0.014** |
|  | 5 a 8 years | 37 (33.9) |  |  | 15 (13.9) |  |  |
|  | > 9 years | 82 (44.6) |  |  | 25 (13.7) |  |  |
|  |  |  |  |  |  |  |  |
| **Depressive symptoms^b^** | Absence | 173 (35.2) | 3.23 | 0.072 | 53 (5.8) | 2.14 | 0.143 |
|  | Presence | 22 (25.3) |  |  | 5 (10.9) |  |  |
|  |  |  |  |  |  |  |  |
| *Pearson Chi-square; ^a^ Non-white skin color (self-reported as Black, Brown, Yellow, or Indigenous); ^b^Depressive symptoms assessed by the Geriatric Depression Scale (GDS); ^†^MVPA trajectory and walking trajectory by the International Physical Activity Questionnaire (IPAQ). | | | | | | | |
